# Efficacy and safety of traditional Chinese medicine for the treatment of pancreatic cancer: An overview of systematic reviews and meta-analyses

**DOI:** 10.3389/fphar.2022.896017

**Published:** 2022-09-01

**Authors:** Jing Wang, Qiuyuan Wang, Peitong Zhang, Ruoqi Zhang, Jie He

**Affiliations:** ^1^ Department of Oncology, Guang’anmen Hospital, China Academy of Chinese Medical Sciences, Beijing, China; ^2^ Graduate School, Beijing University of Chinese Medicine, Beijing, China; ^3^ Department of Orthopaedics, China-Japan Friendship Hospital, Beijing, China

**Keywords:** overview, traditional Chinese medicine, efficacy, pancreatic cancer, meta-analyses

## Abstract

Pancreatic cancer is a highly malignant tumor with poor prognosis. Currently available Western medical management strategies are unable to prolong the survival time and reduce the mortality of patients with pancreatic cancer. Traditional Chinese medicine has achieved promising results in many clinical studies. This systematic review and meta-analysis (SR/MA) aimed to explore the benefits and evaluate the quality of evidence of traditional Chinese medicine-based interventions for preventing and treating pancreatic cancer. A systematic search of eight databases for SRs/MAs of randomized controlled trials on traditional Chinese medicine treatment for pancreatic cancer was conducted (from inception to April 2022). The methodological quality of the SRs/MAs was assessed using AMSTAR 2.0, and the quality of evidence was evaluated using the GRADE guide. Nine SRs/MAs, including 145 randomized controlled trials, were considered eligible for this study. The literature were published between 2014 and 2022. The sample size of randomized controlled trials in the MAs ranged from 336 to 1,989. The methodological quality of the nine studies was critically low. Among the 59 outcome indicators of the nine SRs/MAs, seven, 33, and 19 had moderate-, low-, and critically low-quality evidence, respectively, while high-quality evidence was not identified. The results for the long-term indicators, short-term indicators, and adverse reactions in the SRs/MAs displayed consistencies and differences. In conclusion, the methodological and evidence quality of the current evidence is generally low, highlighting the need for additional focus on implementation processes. Some evidence with moderate quality validated that several specific traditional Chinese medicine were optimum for improving the short-term clinical efficacy. However, more objective and high-quality investigations are warranted to verify the efficacy of traditional Chinese medicine for pancreatic cancer.

## 1 Introduction

Pancreatic cancer (PC) is a highly malignant type of solid tumor. The main clinical manifestations of PC include digestive system symptoms such as anorexia, ascites, and jaundice. Metastases to distant sites, including the lungs, bones, and lymph nodes, can also occur. Owing to its characteristics of the complex tumor microenvironment and rapid progression, PC has a high mortality rate. According to global statistics, from 1990 to 2017, the incidence and mortality of PC have more than doubled (GBD Pancreatic Cancer Collaborators, 2019). Estimates of cancer epidemiological data from the GLOBOCAN database indicate that new cases of pancreatic neoplasms account for 2.5% of all cancer cases, while deaths due to pancreatic neoplasms account for 4.5% of all cancer-associated deaths worldwide. Furthermore, the incidence of PC is almost as high as its mortality in 2018 (432,000 cases and 459,000 deaths) (Bray et al., 2018). Indeed, it is predicted that PC will emerge as the third leading cause of cancer-related deaths, surpassing breast cancer (Ferlay et al., 2016). These data underscore the need to improve treatment strategies for PC.

The limited efficacy of the currently available medical treatments for PC is a key reason underlying the high mortality rate. Chemotherapy as a first-line regimen and rare drug treatments with beneficial effects can result in systemic damage and even fatal side effects, such as bone marrow suppression and liver and kidney toxicity. These effects resemble those of radiotherapy, including radiation pneumonia, radiation enteritis, and skin and mucous membrane damage (Suker et al., 2016; Suker et al., 2018; Philip et al., 2020).

With the advances in traditional Chinese medicine (TCM) research, the clinical value of TCM is being increasingly recognized worldwide. As a complementary and alternative medical approach, TCM has long been utilized by patients in China, especially patients with PC. Indeed, TCM is accepted as a mainstream therapy owing to its curative effects and good safety profiles, in contrast to Western medical treatment (Zhang et al., 2010; Kuo et al., 2018). Accordingly, the widespread application of TCM warrants further exploration to reduce the mortality rate of patients with PC.

To date, there is extensive literature on modern medical evidence-based research methods associated with the prevention and treatment of PC using TCM, including several clinical observational studies and clinical trials. Among them, systematic reviews and meta-analyses (SRs/MAs) provide high-level evidence that can guide clinical decision-making and form an essential basis for clinical workers to formulate diagnosis and treatment guidelines or conduct relevant studies. Although randomized controlled trials (RCTs) of TCM combined with Western medical therapies for PC are searchable in scholarly databases, the evidence remains incomplete, and the results remain controversial. Furthermore, there is yet to be a comprehensive review of SRs/MAs evaluating the use of TCM for PC. Therefore, this study aimed to integrate the specific characteristics and outcome indicators of different SR/MAs using AMSTAR2.0 and GRADE evaluation tools to evaluate the methodological and evidence-level quality of SRs/MAs in order to provide reliable evidence for the clinical prevention and treatment of PC.

## 2 Materials and methods

### 2.1 Inclusion and exclusion criteria

The inclusion criteria were as follows: 1) the research type was an SR/MA of RCTs using TCM for the treatment of PC; 2) patients were diagnosed with PC, regardless of stage, age, sex, race, or nationality; 3) TCM (formula, injection, and patent medicine) was used as the intervention in the experimental group, with or without other therapies such as Western medicine; and 4) at least one efficacy evaluation index was included in the outcome indices, such as clinical symptoms or 1-year survival rate.

The exclusion criteria were as follows: 1) the study participants presented with a category of diseases, including PC, such as digestive cancer; 2) studies were duplicates (duplicates were selected according to their publication time and completeness, and previous duplicates were excluded as subsequent updates were identified); 3) we were unable to obtain the full text or extract the data; 4) the studies were network MAs; and 5) the studies were SRs without MAs.

### 2.2 Literature retrieval

Combinations of subject words and free words were adopted for an extensive search in the CNKI, WanFang, VIP, CBM, PubMed, Web of Science, Embase, and Cochrane Library databases. The search terms included “TCM,” “traditional Chinese medicine,” “Chinese medicine,” “Chinese herb,” “traditional medicine,” “pancreatic cancer,” “pancreatic malignancy,” “pancreatic ductal adenocarcinoma,” “pancreatic carcinoma,” “meta-analysis,” “systematic evaluation,” and “systematic review.” The literature search was independently conducted by two researchers from inception to April 2022.

### 2.3 Literature screening and data extraction

The literature to be screened were imported into Endnote X9 software for literature management. Two reviewers independently performed literature screening and data extraction. Based on the criteria, preliminary screening was conducted by browsing the titles, abstracts, and keywords of the literature and then browsing the full text for further evaluation. Subsequently, the references in the retrieved literature were checked to avoid omissions. The quality of the literature was not evaluated during the screening process. The following essential data were extracted from the eligible studies: authors, publication year, research type, subject, number of RCTs, number of samples, intervention details, methodological characteristics, and funding support. We extracted the pooled effect sizes from these MAs and reported as relative risk (RR), odds ratio (OR), and 95% confidence interval (CI) for dichotomous outcomes, and the mean difference (MD) and 95% CI were used for continuous outcomes. Any disagreement was resolved by discussion between the two reviewers and re-evaluation by a third reviewer.

### 2.4 Evaluation of methodological quality

AMSTAR2.0, an internationally recognized systematic methodological quality evaluation tool (Zhang et al., 2018), was applied to investigate the methodological quality of the included literature. AMSTAR2.0 incorporates 16 items in total. We focused on core items 2, 4, 7, 9, 11, 13, and 15 for evaluating the quality of the literature. Two researchers conducted the evaluations independently. Controversies were resolved by discussion and consultation. When the corresponding literature content matched with a certain item, it was designated “yes,” incomplete matching was designated “partial yes,” and non-matching was designated “no.” The RCTs were initially high-quality studies. When the details of the literature did not conform to a “non-core item,” the literature quality was downgraded. If more than one “non-core item” was not met, the literature was rated as moderate quality. If the literature did not conform to one “core item” with or without a “non-core item,” it was rated as low quality. The literature was considered critically low quality when it was not in accordance with more than one “core item” with or without any “non-core item.”

### 2.5 Evaluation of evidence quality

Evaluation of the quality of evidence as an outcome indicator was performed with reference to the GRADE guidelines (Guyatt G. et al., 2011; Deng et al., 2018). The GRADE guidelines include five aspects: risk of bias (limitations) (Guyatt et al., 2011b), inconsistency (Guyatt et al., 2011c), indirectness (Guyatt et al., 2011d), imprecision (Guyatt et al., 2011e), and publication bias (Guyatt et al., 2011f). The RCTs were initially regarded as high-quality studies. During the evaluation process, the quality of the evidence of the literature was downgraded if any of the aforementioned problems were observed. Ultimately, the quality grade was determined as high, moderate, low, or critically low. Two reviewers performed the assessment independently. Any disagreement was resolved by discussion between the two reviewers or evaluation by a third reviewer.

## 3 Results

**FIGURE 1:**
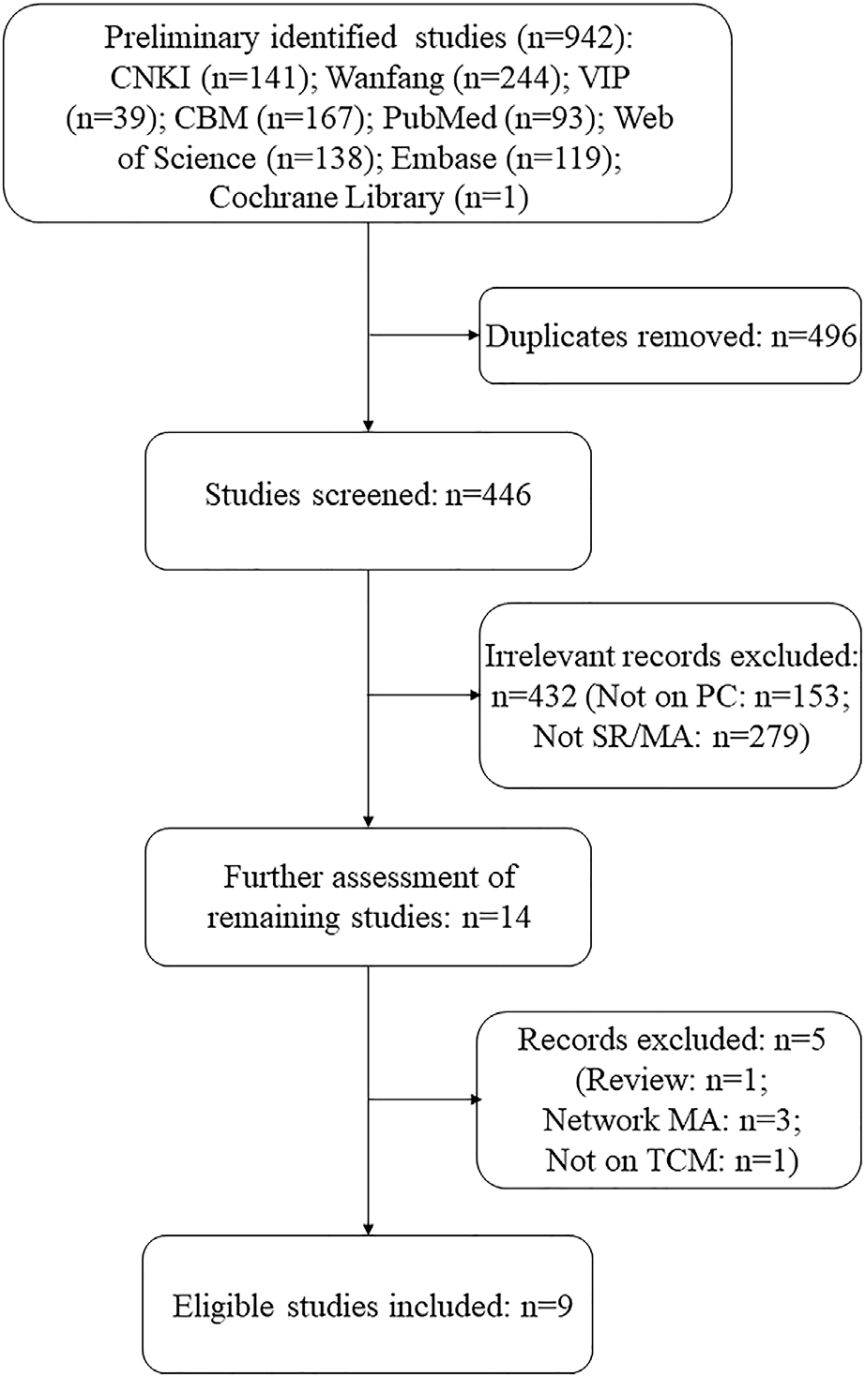
Flowchart of the selection process.

The preliminary search yielded 942 studies, of which 496 were duplicates. After further screening and review, 432 records were excluded. Of the remaining 14 records, five were excluded for the following reasons: one was a review (Gao et al., 2021), one was not on TCM intervention (Song et al., 2011), and three were network MAs (Zhang et al., 2017; Zhang, 2018; Zhu et al., 2018). Finally, nine SRs/MAs of RCTs on TCM interventions for PC met the screening criteria. It is to be noted that only the traditional SRs/MAs that involved quantitative analysis were included in order to obtain more rigorous and uniform evaluation results, so we excluded network MAs and “SRs without MAs.” A flowchart of the selection process is shown in Figure 1.

### 3.1 Characteristics of the included literature

Detailed information on the nine retrieved SRs/MAs is summarized in Table 1. Of the nine studies included, six were in Chinese and three were in English. The research type of seven studies was MA, while the remaining two were SRs/MAs. All literature were published between 2014 and 2022. The number of RCTs in MAs ranged from five to 31, with a minimum sample size of 336 and maximum sample size of 1,989. Three studies involved only patients with PC, three studies involved moderately advanced patients with PC, and three studies included only patients with advanced PC as study participants. In terms of intervention measures, the most common intervention included in the treatment groups in the original studies was Chinese herbal formulas combined with radiotherapy or chemotherapy (6/9, 66.7%). While five studies focused on intervention with Chinese medicine injections combined with radiotherapy or chemotherapy, four studies focused on Chinese patent medicine. To assess the risk of bias of RCTs, six SRs/MAs used the Cochrane Handbook assessment tool, two SRs/MAs used the Jadad scale, and one SR/MA used both the Cochrane assessment tool and Jadad scale to assess quality. In addition, three SRs/MAs performed subgroup analyses, five SRs/MAs performed sensitivity analyses, and seven SRs/MAs used funnel plots to test for publication bias. Regarding funding details, four SRs/MAs reported support from scientific research projects, whereas the remaining studies reported no project support or did not indicate funding information.

**TABLE 1:** Characteristics of the SRs/MAs.

Reference	Type	Number of RCT	Samples	Participant	Interventions		Methodological characteristics				Main TCM type	Funding
					Experimental group	Control group	Quality assessment tool	Subgroup analyses	Sensitivity analyses	Funnel plots		
Lu et al. (2014)	MA	5	336	Moderate to advanced PC	TCM plus chemoradiotherapy	Chemoradiotherapy	Jadad	N	N	Y	Formulas and Chinese patent medicine	NF
Li et al. (2015)	SR/MA	29	1,808	Unresectable PC	TCM plus conventional therapy	Chemotherapy, radiotherapy, transcatheter arterial chemotherapy, high intensity focused ultrasound	Jadad	N	Y	Y	Formulas and injections and Chinese patent medicine	Y
Xie et al. (2017)	MA	12	823	Middle-advanced PC	TCM plus chemotherapy	Chemotherapy	Cochrane & Jadad	N	N	Y	“Strengthening vital qi to eliminate pathogenic factor” formulas and Chinese patent medicine	Y
Wang (2017)	MA	8	617	PC	TCM plus chemotherapy/radiotherapy	Chemotherapy/radiotherapy	Cochrane	N	N	N	Formulas	NF
Deng (2018)	MA	24	1,524	PC	TCM plus chemotherapy	Chemotherapy	Cochrane	Y	Y	Y	Injections	NF
Liu et al. (2019)	MA	16	960	Advanced PC	TCM plus radiochemotherapy	Radiochemotherapy	Cochrane	Y	Y	Y	Kanglaite injection	NF
Ding et al. (2020)	MA	10	531	Advanced PC	TCM plus chemotherapy	Chemotherapy	Cochrane	N	N	Y	Kanglaite injection	Y
Cheng and Cao (2020)	MA	10	597	PC	TCM plus chemotherapy	Chemotherapy	Cochrane	N	Y	Y	Formulas	NF
Hu et al. (2022)	SR/MA	31	1989	Advanced PC	TCM plus chemotherapy	Chemotherapy	Cochrane	Y	Y	N	Formulas and injections and Chinese patent medicine	Y

N, number of RCTs; N, no; Y, yes; NF, not found.

SR, systematic review; MA, meta-analysis.

### 3.2 Assessment of methodological quality

The methodological quality of the nine included studies was rated using the AMSTAR2.0 quality assessment tool. As shown in Table 2, the nine SRs/MAs were all graded as of critically low quality. All literature clearly described each element of the population, intervention, control, and outcome (PICO) principle in parts of the research question and inclusion criteria and indicated that the types of included studies were RCTs. Data extraction was performed by two reviewers to ensure that the process was independent and repeatable. All studies applied appropriate assessment methods to estimate the risk of bias in RCTs and evaluated the probable impact of bias on the results and conclusions. All studies applied appropriate statistical methods to combine the effect sizes of their results. However, eight of the nine studies were not pre-registered or published with a proposal in advance. In addition, none of the SRs/MAs provided funding information for the RCTs. With regard to literature screening and exclusion, all studies described the process of literature screening and quantity of the excluded literature, while only few studies explained the reason for exclusion. However, a list of the excluded literature was not included in the dissertation. As for literature retrieval, only one study (Deng, 2018) adopted a comprehensive and systematic retrieval method, and five studies (Wang, 2017; Deng, 2018; Cheng and Cao, 2020; Ding et al., 2020; Hu et al., 2022) had the literature screened independently and reproducibly by two researchers. To better understand and compare the results, the specific characteristics of the RCTs should be described in detail as denoted by the PICO principles. However, only three studies (Liu et al., 2019; Ding et al., 2020; Hu et al., 2022) presented comprehensive information for each included study, while the descriptions in the other SRs/MAs did not meet the requirements to varying degrees.

**TABLE 2:** AMSTAR 2.0 evaluation.

References	1	2	3	4	5	6	7	8	9	10	11	12	13	14	15	16	Grade
Lu et al. (2014)	Y	N	Y	PY	N	Y	N	PY	Y	N	Y	Y	Y	Y	Y	N	CL
Li et al. (2015)	Y	N	Y	PY	N	Y	N	PY	Y	N	Y	Y	Y	N	Y	Y	CL
Xie et al. (2017)	Y	N	Y	PY	N	Y	N	PY	Y	N	Y	Y	Y	N	Y	N	CL
Wang (2017)	Y	N	Y	PY	Y	Y	N	PY	Y	N	Y	Y	N	Y	N	N	CL
Deng (2018)	Y	N	Y	Y	Y	Y	N	PY	Y	N	Y	Y	Y	N	N	N	CL
Liu et al. (2019)	Y	N	Y	PY	N	Y	N	Y	Y	N	Y	Y	Y	Y	Y	Y	CL
Ding et al. (2020)	Y	N	Y	PY	Y	Y	N	Y	Y	N	Y	Y	Y	Y	Y	N	CL
Cheng and Cao (2020)	Y	N	Y	PY	Y	Y	N	PY	Y	N	Y	Y	Y	N	N	N	CL
Hu et al. (2022)	Y	Y	Y	PY	Y	Y	N	Y	Y	N	Y	Y	Y	Y	N	Y	CL

Y, yes; N, no; PY, partially yes; CL, critically low.

The risk of bias in the included RCTs was not discussed in one study (Wang, 2017), and the impact of heterogeneity between studies on the outcomes was not discussed in four studies (Li et al., 2015; Xie et al., 2017; Deng, 2018; Cheng and Cao, 2020). Five studies (Lu et al., 2014; Li et al., 2015; Xie et al., 2017; Liu et al., 2019; Ding et al., 2020) assessed publication bias and discussed its effects on the results.

### 3.3 Evaluation of evidence quality

A total of 59 outcome indicators were included in the nine SRs/MAs. The evidence body, composed of outcome indicators, was evaluated according to the GRADE evaluation guide. Among the 59 outcome indicators, seven had moderate-quality, 33 had low-quality, and 19 had critically low-quality evidence. No high-quality evidence was identified. The evaluation results are listed in Table 3. The long-term outcome indicators included three items of low-level evidence and two items of critically low-level evidence. The efficacy rate, which was the most widely reported outcome indicator, included five outcomes with low-grade evidence and one outcome with moderate-grade evidence.

**TABLE 3:** GRADE evaluation results.

References	Indicators	Limitations	Inconsistency	Indirectness	Imprecision	Publication bias	Quality
Lu et al. (2014)	Clinical efficacy	↓	-	-	-	↓	L
Li et al. (2015)	6-month survival rate	↓	↓	-	-	↓	CL
	1-year survival rate	↓	-	-	-	↓	L
	Objective response rate	↓	-	-	-	↓	L
	DCR	↓	↓	-	-	↓	CL
	QOL	↓	↓	-	↓	↓	CL
	Clinical benefit response	↓	-	-	-	↓	L
	Grade I leukopenia	↓	↓	-	-	↓	CL
	Grade III–IV leukopenia	↓	-	-	-	↓	L
	Grade I–IV thrombocytopenia	↓	-	-	↓	↓	CL
	Grade III–IV thrombocytopenia	↓	-	-	↓	↓	CL
	Grade III–IV nausea and vomiting	↓	-	-	-	↓	L
Xie et al. (2017)	Recent clinical efficacy	↓	-	-	-	↓	L
	Physical condition	↓	-	-	-	↓	L
	1-year survival rate	↓	-	-	-	↓	L
	Incidence of leukopenia	↓	-	-	-	↓	L
Wang (2017)	Complete remission rate	↓	-	-	↓	↓	CL
	Disease efficacy rate	↓	-	-	-	↓	L
Deng (2018)	Clinical efficacy rate	↓	-	-	-	-	M
	Clinical benefit rate	↓	-	-	-	-	M
	Myelosuppression	↓	-	-	-	-	M
	Pain symptom relief	↓	-	-	-	↓	L
	KPS improvement	↓	-	-	-	-	M
Liu et al. (2019)	1-year survival rate	↓	-	-	-	↓	L
	2-year survival rate	↓	-	-	↓	↓	CL
	CR	↓	-	-	-	↓	L
	PR	↓	-	-	-	↓	L
	SD	↓	-	-	↓	↓	CL
	PD	↓	-	-	-	↓	L
	Overall response rate	↓	-	-	-	-	M
	DCR	↓	-	-	-	-	M
	CEA,CA19-9	↓	-	-	↓	↓	CL
	QIR	↓	-	-	-	↓	L
	PRR	↓	-	-	-	↓	L
	WGR	↓	-	-	-	↓	L
	Adverse events	↓	-	-	-	↓	L
Ding et al. (2020)	Efficacy rate	↓	-	-	-	↓	L
	QOL improvement rate	↓	-	-	-	↓	L
	Pain improvement rate	↓	-	-	-	↓	L
	Weight improvement rate	↓	-	-	-	↓	L
	Safety indexes	↓	-	-	↓	↓	CL
Cheng and Cao (2020)	Short-term efficacy	↓	-	-	-	↓	L
	CA19-9	↓	-	-	↓	↓	CL
	QOL	↓	-	-	-	↓	L
	Myelosuppression rate	↓	-	-	-	↓	L
	Gastrointestinal adverse reactions	↓	-	-	-	↓	L
Hu et al. (2022)	Objective response rate	↓	-	-	-	-	M
	DCR	↓	-	-	-	↓	L
	QOL	↓	↓	-	↓	↓	CL
	CA19-9	↓	↓	-	↓	↓	CL
	CEA	↓	↓	-	↓	↓	CL
	Leukopenia	↓	-	-	-	↓	L
	Decreased hemoglobin	↓	-	-	-	↓	L
	Thrombopenia	↓	-	-	-	↓	L
	Myelosuppression	**↓**	-	-	**↓**	↓	CL
	Nausea and vomiting	↓	-	-	**↓**	↓	CL
	Gastrointestinal reaction	↓	-	-	-	↓	L
	Liver dysfunction	↓	-	-	**↓**	↓	CL
	Hair loss	↓	-	-	**↓**	↓	CL

↓, downgrade; -, not observed; M, moderate; L, low; CL, critically low.

DCR, disease control rate; QOL, quality of life; CR, complete response; QIR, QOL, improvement rate; PR, partial response; SD, stable disease; PD, progressive disease; CEA, carcinoembryonic antigen; CA19-9, carbohydrate antigen 19-9; PRR, pain relief rate; WGR, weight gain rate; KPS, Karnofsky performance score.

Quality of life (QOL)-related indicators were dominated by low-quality evidence, while the improvement in tumor inspection indicators was critically low. In terms of alleviating adverse reactions to Western medicine treatments, the evidence body quality was mainly low and extremely low, except for one outcome with moderate-quality evidence. All evidence bodies were downgraded for limitations (88.14% for publication bias, 27.12% for imprecision, and 11.86% for inconsistency).

#### 3.3.1 Long-term indicators

Survival rate was defined as the main long-term outcome index for evaluating the effects of TCM treatment and constituted an objective evaluation indicator. Three SRs/MAs (Li et al., 2015; Xie et al., 2017; Liu et al., 2019) were used to evaluate the survival rates. In the study by Li et al. (2015), the pooled RRs of the 6-month and 1-year survival rates of the TCM treatment group were 1.58 (95% CI = 1.05–2.37, *p* = 0.03) and 1.85 (95% CI = 1.49–2.31, *p* < 0.00001), respectively. In combination with the results of heterogeneity evaluation, it was concluded that the 1-year survival rate of patients with unresectable PC was significantly improved in the TCM and Western medicine treatment groups when compared with the Western medicine treatment group. Xie et al. (2017) concluded that the 1-year survival rate after TCM treatment, consisting of strengthening health and eliminating pathogenic herbs, combined with chemotherapy was significantly higher than that of the chemotherapy group (RR = 1.60, 95% CI = 1.16–2.21, *p* = 0.004). Liu et al. (2019) calculated effect sizes by combining the 1-year survival rate (OR = 2.58, 95% CI = 1.12–5.93, *p* = 0.03) and 2-year survival rate (OR = 1.59, 95% CI = 0.49–5.15, *p* = 0.44) in the included studies. They observed significant between-group differences in the 1-year survival rates but not in the 2-year survival rates.

#### 3.3.2 Short-term indicators

The short-term outcome indicators in the nine SRs/MAs included objective response rate, overall response rate, disease control rate (DCR), clinical benefit rate, complete response (CR), disease response rate, pain symptom relief, weight improvement, QOL improvement, and changes in levels of biomarkers such as carcinoembryonic antigen (CEA) and carbohydrate antigen 19-9 (CA19-9). Li et al. (2015) and Liu et al. (2019) concluded that TCM formulas and Kanglaite injection combined with Western medicine treatment significantly improved the response rate and DCR of patients with PC. Li et al. (2015) and Deng (2018) reported that TCM formulas and injections improved the clinical benefit. Six studies combined the effect size of the clinical efficacy rate (Lu et al., 2014; Wang, 2017; Xie et al., 2017; Deng, 2018; Cheng and Cao, 2020; Ding et al., 2020) and observed significant differences between the experimental and control groups. Two studies (Li et al., 2015; Hu et al., 2022) reported the objective response rate as an outcome indicator. Two studies (Wang, 2017; Liu et al., 2019) reported a complete remission rate. Among these studies, Wang (2017) reported a negative conclusion: there was no significant difference in the complete remission rate between TCM combined with chemotherapy and chemotherapy alone intervention groups. Liu et al. (2019) also reported significant differences in the partial response (PR) and progressive disease (PD) indices but not in the stable disease (SD) index.

Five studies (Li et al., 2015; Liu et al., 2019; Cheng and Cao, 2020; Ding et al., 2020; Hu et al., 2022) reported efficacy differences in QOL improvement. Li et al. (2015) and Hu et al. (2022) compared the pooled analysis of QOL results using the expression of RR and MD. Li et al. (2015) demonstrated the potential benefit of TCM combined with Western medicine on QOL improvement compared to Western medicine, but the difference only existed when the RR was pooled for the measurement data, whereas the difference was not statistically significant when the MD effect size was pooled for the count data. In the results of the study by Hu et al. (2022), high heterogeneity (I^2^ = 78%) was observed in the QOL continuous data. In addition to these indicators, there were other pieces of evidence that Western medicine alone improved the short-term outcomes in an inferior manner to TCM combined with Western medicine. One study (Deng, 2018) reported improvements in the Karnofsky performance score (KPS) with TCM injections, and another study (Xie et al., 2017) reported improvements in physical performance with TCM formulas. Three studies (Deng, 2018; Liu et al., 2019; Ding et al., 2020) reported pain symptom relief. Two studies (Liu et al., 2019; Ding et al., 2020) reported differences in body weight improvement. In terms of laboratory indices, three studies (Liu et al., 2019; Cheng and Cao, 2020; Hu et al., 2022)reported efficacy differences in the CEA and CA19-9 levels.

#### 3.3.3 Adverse reactions

Myelosuppression and leukopenia are two frequent adverse reactions in patients with PC undergoing chemotherapy. Accordingly, data on leukopenia (5/9 SRs/MAs) (Li et al., 2015; Xie et al., 2017; Liu et al., 2019; Ding et al., 2020; Hu et al., 2022) and myelosuppression (5/9 SRs/MAs) (Deng, 2018; Liu et al., 2019; Cheng and Cao, 2020; Ding et al., 2020; Hu et al., 2022) were mostly pooled for analysis as outcome measures. Two studies (Liu et al., 2019; Ding et al., 2020) reported that Kanglaite injection combined with radiotherapy and chemotherapy ameliorated leukopenia. In contrast, Xie et al. (2017) observed no significant difference in the amelioration of leukopenia with or without the use of TCM for strengthening the body and eliminating pathogenic factors during chemotherapy. Li et al. (2015) reported a difference in grade III–IV leukopenia but not in grades I–IV. Hu et al. (2022) found that TCM combined with chemotherapy reduced the incidence of leukopenia. Thrombocytopenia, liver and kidney toxicity, gastrointestinal adverse reactions, and skin and mucous membrane damage are common side effects of radiotherapy and chemotherapy. Of the nine SRs/MAs on PC treatment with TCM, four (Li et al., 2015; Liu et al., 2019; Ding et al., 2020; Hu et al., 2022) reported thrombocytopenia as an outcome indicator. Li et al. (2015) concluded that there was no significant difference in the improvement of thrombocytopenia between the control and experimental groups. In addition, four SRs/MAs (Li et al., 2015; Liu et al., 2019; Cheng and Cao, 2020; Hu et al., 2022) reported significant differences in the improvement of gastrointestinal adverse reactions, while Hu et al. (2022) obtained negative results on nausea and vomiting. In addition, Hu et al. (2022) also observed no difference in the improvement of myelosuppression, liver dysfunction, and hair loss when combining TCM with chemotherapy as interventions. Two SRs/MAs (Liu et al., 2019; Ding et al., 2020) reported that Kanglaite injection combined with chemotherapy relieved renal damage. One study (Ding et al., 2020) demonstrated that Kanglaite injection relieved the hepatotoxicity of chemotherapy and had no benefit in chemotherapy-related nausea, vomiting, or neurotoxicity. Liu et al. (2019) found no significant differences in hepatotoxicity, neurotoxicity, anemia, nausea, vomiting, diarrhea, rash, weakness, fatigue, and anorexia with the combined use of radiochemotherapy and Kanglaite injection compared to the radiochemotherapy group.

## 4 Discussion

Patients with PC have poor prognosis, short lifespan, and poor QOL due to symptoms such as pain, ascites, and anorexia. Despite extensive research, effective therapeutic methods are lacking, and current treatments fail to address the high mortality rate. Accordingly, there is an urgent need to identify alternative therapeutic options. In China, TCM is widely accepted and adopted by patients with PC. A systematic review summarizes several clinical studies on applying plants in the treatment of PC, and the research elucidates that several herbs, extracts, and formulas in different clinical studies can lengthen the median progression-free survival, median overall survival, and overall survival rate and can improve the symptoms of PC patients (Triantafillidis et al., 2022). Evidence suggests that TCM improves the integrated treatment effects and QOL of patients. However, the clinical results remain controversial. Thus, our research focused on the efficacy and safety of TCM reported in SRs/MAs of RCTs to identify robust evidence for the clinical application of TCM and expand future research on PC.

Our study is the first overview to summarize the quality of all published Chinese and English SRs/MAs of RCTs assessing TCM as a treatment for PC. Using comprehensive retrieval and evaluation of SRs/MAs, we identified the efficacy of TCM in the prevention and treatment of PC in many respects. The most valuable findings of this overview are the seven items of evidence with moderate quality. Four of them are from the study of Deng (2018). The results in this study indicated traditional Chinese herb injections combined with chemotherapy could improve the clinical efficacy rate [OR = 2.3491, 95% CI = (1.8468–2.9878), *p* = 0.001], clinical benefit rate [OR = 2.4746, 95% CI = (1.9023–3.2191), *p* = 0.001], and KPS score [OR = 3.0944, 95% CI = (2.2452–4.2648), *p* = 0.0000] and reduce myelosuppression [OR = 0.3882, 95% CI = (0.2752–0.5475), *p* = 0.0001]. In the subgroup analysis, the effect value of *Brucea javanica* oil emulsion injection was found optimum in improving the clinical efficacy rate [OR = 5.4444, 95% CI = (0.9175–32.3059)] and clinical benefit rate [OR = 4.2, 95% CI = (0.6982–25.2641)] and in reducing myelosuppression [OR = 0.2308, 95% CI = (0.0469–1.1346)]. For KPS score improvement, the compound Kushen injection was found to be the most effective [OR = 4.0741, 95% CI = (2.1549–7.7025)]. The other two items of evidence with moderate quality stem from the study of Liu et al. (2019). This study included 16 trails with 960 patients and indicated that Kanglaite injection combined with radiochemotherapy could improve the overall response rate [OR = 2.16, 95% CI = (1.58–2.94), *p* < 0.00001] and DCR [OR = 2.5, 95% CI = (1.84–3.38), *p* < 0.00001]. The last item comes from the research of Hu et al. (2022). They demonstrated that objective response rate could be ameliorated after the application of TCM and chemotherapy in advanced PC patients [RR = 1.64, 95% CI = (1.43–1.88), *p* < 0.00001]. It is to be noted that in order to determine which traditional Chinese herb or herb combination contributed the most to the outcome, they performed a subgroup analysis of the TCM components listed in each included study. They found six herbs (*Atractylodes macrocephala*, *Glycyrrhiza glabra*, *Astragalus mongholicus*, *Codonopsis pilosula*, *Poria cocos*, and *Pinellia ternata*) and different multiple combinations had significant RRs and little heterogeneity. Simultaneously, the subgroup analysis results also showed that TCM combined with chemotherapy increased the objective response rate when KPS score <70, while the difference of the objective response rate was not related to oral or intravenous administration. The main ingredients in TCM injections are shown in Supplementary Material S1.

The seven items of evidence with moderate quality are mostly derived from the studies related to traditional Chinese herb injections. This may be due to the more standard and unified characteristics of Chinese herb injections than TCM formulas. As one of the TCM preparations in the adjuvant treatment of tumors, the anticancer efficacy of traditional Chinese herb injections has been tested in clinical practice for many years, and the pharmacological effects of its components have been fully confirmed in considerable studies. Among the TCM interventions corresponding to these superior evidence, *Brucea javanica* oil emulsion injection is the fruit of *Brucea javanica (L.) Merr.*, containing oleic, linoleic, palmitic, arachidonic, and stearic acid (Chen et al., 2022). Animal experiments and clinical trials have confirmed that these components have inhibitory effects on malignant tumors (Ma et al., 2013). In addition, *Brucea chinensis* oil can be used as the main component of the microemulsion to form a new formulation of docetaxel for intravenous administration, and the microemulsion of docetaxel shows superb pharmacokinetic characteristics, such as longer half-time (Ma et al., 2013). The compound Kushen injection is an extract of *Sophora flavescens* and *Smilax glabra* and contains oxymatrine and matrine (Tian et al., 2007). The main components of compound Kushen injection were measured by fingerprints and other methods in different studies, and the results were consistent (Xu et al., 2011; Jin et al., 2018). Oxymatrine, oxysophocarpine, matrine, and sophocarpine were all determined. Experiments manifest that compound Kushen injection plays a role in inhibiting the proliferation of tumor stem cells (Xu et al., 2011), tumor growth (Zhao et al., 2014), and tumor metastasis (Dai et al., 2007). Kanglaite injection is extracted from *Coix lacryma-jobi*, and its main active ingredient is triglyceride containing four fatty acids (Wang et al., 2017). Kanglaite injection can promote tumor cell apoptosis (Yuan et al., 2004), inhibit cell proliferation and tumor growth *in vivo* (Fang et al., 2020), and also enhance the effect of chemotherapy when combined with chemotherapy drugs such as paclitaxel (Wang et al., 2017).

The main components of TCM formulas usually varied differently in clinical practice. Therefore, high heterogeneity was observed in SRs/MAs of TCM formula research, leading to inferior grade of evidence. In the study of Hu et al. (2022), a new herbal subgroup analysis was applied, and several herbs with the highest contribution were obtained. Summarizing the abovementioned evidence, we can conclude that the following Chinese herbs are the most promising and important TCM for improving PC treatment efficacy and prolonging survival: *Brucea javanica*, *Sophora flavescens*, *Smilax glabra*, *Coix lacryma-jobi*, *Atractylodes macrocephala*, *Glycyrrhiza glabra*, *Astragalus mongholicus*, *Codonopsis pilosula*, *Poria cocos*, and *Pinellia ternate.*

From the results of the evidence, it is not difficult to observe the advantages of traditional Chinese herb injections in the efficacy of PC patients. In order to conduct a horizontal comparison between TCM injections, we further searched all the Chinese and English databases to obtain network meta-analyses on TCM injection treatment of PC. A total of three network meta-analyses were retrieved (Zhang et al., 2017; Zhu et al., 2018; Wang et al., 2022). It is worth mentioning that we searched for all network meta-analyses on TCM treatment of PC and also only found these three research studies. We excluded one Chinese literature which could not draw accurate conclusions due to serious problems in methods and results. A network meta-analysis included 22 RCTs involving 1,329 patients (Zhang et al., 2017). They compared the efficacy of nine traditional Chinese medicine injections, and the results exhibited that compound Kushen, Kangai, and Kanglaite injections combined with chemotherapy possessed higher probability of ameliorating the performance of PC, and Aidi injection combined with chemotherapy was significantly effective in relieving leucopenia. The efficacy advantage of Aidi injection was also confirmed in the other network meta-analyses (Wang et al., 2022). They compared the effects of eight Chinese herb injections and emphasized that Aidi injection could significantly improve clinical efficacy [OR = 0.34, 95% CI = (0.16–0.74)], manifested in remission of chemotherapy-induced leukopenia and thrombocytopenia [OR = 5.65, 95% CI = (1.18–28.13)]. Among the seven higher-quality pieces of evidence we analyzed, the improvement of KPS score by compound Kushen injection was further supported by the results of network meta-analyses.

There are also some discrepancies between the included SRs/MAs. For example, Ding et al. (2020) reported no significant differences in the incidence of nausea and vomiting after treatment with Kanglaite injection combined with chemotherapy compared to the chemotherapy group, whereas Liu et al. (2019) inferred different conclusions. Nevertheless, some conclusions drawn from the available SRs/MAs are not sufficiently convincing, either from methodological or evidence quality perspectives. In this regard, clinical research on TCM for PC treatment remains problematic.

The SRs/MAs included in this overview had several methodological defects. Some studies poorly described the basic characteristics of the included studies and did not clearly present the information according to the PICO principles of the included RCTs, including disease stage, sample size, duration of intervention, and intervention dose, thus failing to reflect the commonalities and differences between the studies. The evaluators could not conclude whether the inclusion period of the studies was appropriate or whether there was clinical heterogeneity among studies, which precluded further evaluation of the quality of the results for clinical decision-making. Furthermore, formulating a protocol before study implementation enables early examination of research methods, which is a critical step in implementing an SR/MA and can help reduce the possible risk of bias in trials. However, the included SRs/MAs lacked this feature. Moreover, the funding details of the RCTs, conflicts of interest, and funding sources of SRs/MAs should be explained to help reviewers determine the potential influence of corporations or sponsors on data collection, interpretation, and objectivity of the conclusions. Additionally, literature screening and data extraction should be independently conducted by two or more researchers to ensure repeatability of the entire process and avoid mistakes. In addition, all published databases, clinical trial registration websites, and dissertation databases should be included in the scope of the literature search, as well as the references of the included studies, reviews in related fields, and literature in gray areas. Relevant reasons should be listed when excluding the literature. Some SRs/MAs in this overview included a flowchart of the search process and reasons for exclusion. However, none of the SRs/MAs provided an exclusion list. Furthermore, the Cochrane risk of bias assessment tool and Jadad scale were used to evaluate bias in the methodology of the included RCTs. Some studies excluded certain RCTs owing to the high risk of bias during literature screening. However, this method was not recommended in the AMSTAR 2.0 guide. Some researchers did not consider quality evaluation of RCTs and only summarized the scores of methodology assessment to assess the risk of bias without a detailed description. Thus, the combination of results and further evaluation might have been affected. It is to be noted that although only one study did not measure publication bias, some of the measured studies only measured the publication bias of individual indicators such as the primary outcome indicator, which could reduce the reliability of the results. Moreover, bias of included studies, heterogeneity between studies, and publication bias are common factors that affect the internal and external validity of the results. However, some studies did not fully discuss these factors, and the conclusions were not drawn rigorously.

We employed the GRADE scoring standard to evaluate the quality of the evidence body of all studies to assess the power of whether the effect estimates accurately reflected the real situation. The quality of the available evidence from SRs/MAs on the treatment of PC with TCM was poor. Most of the evidence was of low-level or critically low-level quality. Moderate-quality evidence accounted for a small proportion of studies. The main reasons for downgrading evidence were limitations and publication bias. Most of these limitations were attributed to the high risk of bias in the original studies. The RCT quality evaluation results recorded in the SRs/MAs indicated that most of the original studies had problems with critical processes such as randomization, concealment, and blinding. In this regard, the imprecision or error of the original research directly affected the quality of the research and credibility of the results. Publication bias not only mostly occurred in small-sample studies but was also observed in large-sample studies. When the number of original studies or the sample size was small, the existence of publication bias was more likely if there was a lack of measurement results for publication bias. Inconsistency and imprecision were also more common in low-quality bodies of evidence. Inconsistency was evaluated using the standard line of I^2^ as 50%. If the heterogeneity among the RCTs included in the SRs/MAs was high, the body of evidence was inconsistent. However, some SRs/MAs did not have a body of evidence that was evaluated as inconsistent but demonstrated varying degrees of elevation of I^2^. These data indicated that heterogeneity was common among the included studies. Furthermore, combined with the methodological quality evaluation results, only half of the SRs/MAs discussed the source of heterogeneity and its impact on the results. The remaining studies did not mention or explain the heterogeneity. Investigators may not be sufficiently concerned or aware of the effect of heterogeneity on studies. High heterogeneity of the included studies implied that the results of the studies were prone to conflict, and there was insufficient confidence to draw consistent conclusions. In addition, a small sample size could contribute to the inaccuracy of the body of evidence, which in turn would result in a downgrade of evidence quality. Of note, all the safety indicators were evaluated when TCM was used in conjunction with Western medicine. In this study, it was difficult to distinguish and identify the adverse reactions from TCM, and this aspect should be considered in subsequent studies.

### 4.1 Limitations

This study was not pre-registered. Moreover, the evaluators in this overview studied all the evaluation tools together, but the evaluation was based on personal understanding. As such, the evaluation of the results was partly subjective.

### 4.2 Conclusion

Currently, some evidence demonstrates the potential benefits of TCM combined with Western medicine. In this overview, we verified that the methodological and evidence quality of current SRs/MAs for the treatment of PC with TCM is generally low, partly owing to the poor quality and insufficient quantity of the RCTs included. Moreover, we found out some valuable evidence with moderate quality, which validated the short-term clinical efficacy of several specific TCM. Specifically, the implementation of RCTs was not strict. Researchers should strengthen their understanding of methodological guidelines, especially the importance and significance of methodological concepts such as bias and heterogeneity. Therefore, more high-quality, large-sample RCTs, and SRs/MAs are required to produce more persuasive evidence for promoting the application and implementation of TCM for PC treatment. Collectively, this step will ensure translation to real-world clinical treatment and increase the credibility and authenticity of the results.

## Data Availability

The original contributions presented in the study are included in the article/Supplementary Material. Further inquiries can be directed to the corresponding author.
