# Supplementary material for: Efficacy and safety of traditional Chinese medicine for the treatment of pancreatic cancer: An overview of systematic reviews and meta-analyses
Source: Front Pharmacol. 2022 Sep 1;13:896017. doi: 10.3389/fphar.2022.896017 (PMC9475193; doi:10.3389/fphar.2022.896017)
Supplement: Supplementary file 1 [file Table1.docx]

Supplementary 1. Characteristics of the seven items of evidence

| **References** | **TCM Interventions** | **Main composition** | **Subject** | **Treatment option** | **Evidence** | **OR/RR** | **95%CI** | **Heterogeneity** |
| --- | --- | --- | --- | --- | --- | --- | --- | --- |
| Deng (2018) | Brucea javanica oil emulsion injection | *Brucea javanica* | PC | Combined with chemotherapy | Clinical efficacy rate improvement | 5.4444 | [0.9175-32.3059] | NF |
|  |  |  |  |  | Clinical benefit rate improvement | 4.2 | [0.6982-25.2641] | NF |
|  |  |  |  |  | Myelosuppression reduction | 0.2308 | [0.0469-1.1346] | NF |
|  | Compound Kushen injection | *Sophora flavescens*, *Smilax glabra* | PC | Combined with chemotherapy | KPS score improvement | 4.0741 | [2.1549-7.7025] | 0% |
| Liu et al. (2019) | Kanglaite injection | *Coix lacryma-jobi* | Advanced PC | Combined with radiochemotherapy | Overall response rate improvement | 2.16 | [1.58-2.94] | 0% |
|  |  |  |  |  | DCR improvement | 2.5 | [1.84-3.38] | 0% |
| Hu et al. (2022) | Chinese herb medicine | *Atractylodes macrocephala, Glycyrrhiza glabra, Astragalus mongholicus, Codonopsis pilosula, Poria cocos, and Pinellia ternata* | Advanced PC | Combined with chemotherapy | Objective response rate improvement | 1.64 | [1.43-1.88] | 0% |

KPS, Karnofsky performance score; DCR, disease control rate; NF: not found
